# Effect of Zishen Jiangtang Pill, a Chinese Herbal Product, on Rats with Diabetic Osteoporosis

**DOI:** 10.1155/2018/7201914

**Published:** 2018-02-12

**Authors:** Huilin Li, Shufang Chu, Hengxia Zhao, Deliang Liu, Xuemei Liu, Xi Qu, Jianpin Chen, Zengyin Li, Jinhua Li

**Affiliations:** Shenzhen Traditional Chinese Medicine Hospital, Guangzhou University of Chinese Medicine, Shenzhen 518033, China

## Abstract

Diabetic osteoporosis (DO) is a complication of diabetes. Zishen Jiangtang Pill (ZJP) is a Chinese herbal product which has been used in clinic to maintain blood glucose level and bone density for decades. However, the evidence about its mechanism on diabetes and osteoporosis is still unknown. The aim of this study is to investigate therapeutic effect of ZJP on DO in streptozotocin- (STZ-) induced rats. Rats were randomly assigned to 4 groups: one control group (CON), one model group (MOD), and two ZJP treatment groups (1.5 and 3.0 g/kg/d). All rats were treated for 8 weeks. Results showed that ZJP decreased the blood glucose level during OGTT and prevented the changes of FBG and Fins. Similarly, ZJP inhibited the changes of BCa, P, TRACP-5b, CTX-1, BALP, and BGP and the reduction of BMD. In parallel, 1H-NMR metabolomic studies showed that ZJP significantly altered the metabolic fingerprints of blood and urine level. These findings suggest that ZJP can effectively improve glucose metabolism, abnormal bone metabolism, and metabolic disorders in DO rats, which may be a useful alternative medicine for DO therapy.

## 1. Introduction

Diabetes is a devastating and life-altering disease, which causes many complications and comorbidities. Peripheral vascular, renal, cardiovascular, and neurologic comorbidities are the four major chronic complications that affect total expenditures for diabetes management [1]. Type 2 diabetes (T2DM) constitutes about 90% of all diabetes cases worldwide [2]. Increasing evidences support that T2DM is due to impaired secondary signaling to the binding of insulin to its receptor. Insulin induced the activation of signaling proteins, such as insulin receptor substrate- (IRS-) 1 and IRS-2, and is further attenuated in many tissues, including liver, skeletal muscle, kidney, and bone [3–5]. Moreover, the endocrine and metabolic alterations of T2DM could cause disorders in different pathways related to forming and maintaining of bone [6]. Thus, T2DM can bring several serious consequences, in particular, osteoporosis [7, 8]. Although there are many factors that could increase the risk of osteoporosis including genetic, hormonal, and specific pharmacological therapies [9], diabetes became one of the common causes for osteoporosis.

Diabetic osteoporosis (DO) has been increasingly recognized as an important complication of diabetes [10, 11], the reason of which is resulting from reduced bone mineral content due to the abnormal levels of sugar, protein, fat, and microelements [12]. It has been reported that no difference was observed in the antifracture efficacy of bisphosphonates and raloxifene between patients with diabetes and nondiabetic controls or between patients with type 1 diabetes (T1DM) and T2DM [13], indicating that diabetic patients may receive treatment for osteoporosis in the same way as nondiabetic patients. Bisphosphonates are effective treatment methods for preventing fractures in glucocorticoid-induced osteoporosis. Currently, antiresorptive therapies was evaluated in diabetic patients. Strontium ranelate has also been reported to reduce bone resorption and decreases fracture risk [14, 15]. However, based on existing rodent models, the observation supported crosstalk between the skeleton and energy metabolism, suggesting that osteoporosis therapies may have effects on glucose metabolism, and risk of diabetes should be evaluated [16]. In particular, osteocalcin has beneficial effects on glucose metabolism in animal model [17]. Thus, it is important to look for drugs that are effective and have low side effects in the treatment of DO.

Recently, a number of patients also may choose alternative therapeutic approaches, such as traditional Chinese medicine (TCM). Zishen Jiangtang Pill (ZJP), a Chinese herbal medicine, was comprised of 14 herbs such as Astragalus, Radix Rehmanniae Recens, Radix Rehmanniae Praeparata, Schisandra, Herba Epimedii, Rhizoma Cibotii, and Plastrum Testudinis, which have been used for treatment of diabetic patients in clinic for many years. ZJP possessed the efficacies of tonifying qi and yin, nourishing kidney and bones. Pharmacological studies have reported that ZJP could regulate plasma glucose and lipid levels [18]. Moreover, we found that ZJP inhibited the adipogenic differentiation of mouse bone marrow mesenchymal stem cells (BMSCs) showing the potential role in antiosteoporosis [14, 15]. However, the molecular mechanism of ZJP in DO remains unclear. In the current study, we hypothesize that ZJP could improve DO by regulating glucose and bone metabolism by using a diabetes model of rat. We examined whether ZJP would regulate the glucose and bone metabolism and performed 1H-NMR based urinary and blood metabonomic studies in ZJP treated DO rats.

## 2. Materials and Methods

### 2.1. Animal

Male Sprague-Dawley rats (*n* = 105, 190–220 g, 7-8 weeks of age) were obtained from Experimental Animal Center of Guangdong Province. Animals were maintained in a specific pathogen-free laboratory with regular 12/12 hours light/dark cycles with the average temperature of 25°C and humidity conditions. All animal procedures were approved by the Animal Care and Use Committee at Experimental Animal Center of Guangdong Province and were conducted in accordance with the policies of the Ethics Committee for Animal Research.

### 2.2. Establishment of Rat Diabetes Model and Drug Administration

The animals were allowed to acclimatize for a week before beginning experiments. Rats were randomly assigned to 4 groups: (1) control group (CON) (injection with citrate buffer, intraperitoneally) (*n* = 10); (2) model group (MOD) (injection with streptozotocin (STZ), 55 mg/kg, intraperitoneally) (*n* = 10); (3) model group with 3.0 g/kg/d of ZJP orally (MOD + H-ZJP) (*n* = 10); (4) model group with 1.5 g/kg/d of ZJP orally (MOD + L-ZJP) (*n* = 10). ZJP was obtained from Shenzhen Traditional Chinese Medicine Hospital (Shenzhen, China). STZ was purchased from Sigma-Aldrich (St Louis, MO, USA).

Rats in diabetic and drug-treated diabetic groups were intraperitoneally injected with STZ on one week to induce diabetes. Rats in control group were fed with standard chow and water ad libitum and injected with citrate vehicle alone. After one week of STZ injection, rats were fasted for 12 hours; then venous blood was collected to examine the glucose levels. The glucose levels over 16.7 mmol/L were considered diabetic and selected for further studies. All rats were provided with a vehicle control or drug (ZJP) for 8 weeks after STZ injection. Rats were placed in metabolic cages to collect 24-hour urine after 8 weeks of oral gavage administration of drug. After the 24-hour urine was collected, oral glucose tolerance test (OGTT) was performed. Rats were fed with standard chow and water ad libitum for another week after OGTT; then all the animals were fasted for 12 hours and anesthetized using 2% (w/v) pentobarbital sodium (50 mg/kg, Solarbio Science & Technology, Beijing, China) via intraperitoneal injection. After abdominal aorta blood sampling, rats were killed. The serum was separated by centrifugation at 3000 rpm and stored at −80°C until analyzed. The bones were collected from each animal and dissected with care being taken to protect the periosteum. Each bone was individually wrapped in ddH2O-soaked gauze and stored at −80°C until analyzed.

### 2.3. Oral Glucose Tolerance Test (OGTT)

After overnight fasting, all animals received glucose (3 g/kg) orally. Serum glucose was measured by using glucometer at 0, 0.5, 1.0, and 2.0 hours after glucose intake.

### 2.4. Examination of Glucose and Bone Metabolism

The levels of glycosylated hemoglobin (HbAlc), fasting insulin (Fins), tartrate-resistant acid phosphatase-5b (TRACP-5b), bone specific alkaline phosphatase (BALP), type I procollagen (PINP), and osteocalcin (BGP) were quantified using Quantikine ELISA kit (RD, USA). The experiment was carried out according to user's menu from the manufacturer. Plates were read using an ELISA reader (Hercules, CA, USA) at 450 nm. The concentrations of HbAlc, Fins, TRACP-5b, BALP, PINP, and BGP were calculated using standard calibration curve prepared by using serial dilutions of the standard provided with the kit. The levels of fasting blood glucose (FBG), blood calcium (BCa), phosphorus (P), and urinary calcium (UCa) were examined by using Hitachi fully automatic chemistry analyzer (Beijing Tailin Oriental Trading Company, Beijing, China).

### 2.5. Measurement of Bone Mineral Density (BMD)

Total bone mineral density (T-BMD), spine bone mineral density (S-BMD), and left thigh bone mineral density (LT-BMD) were measured by using dual-energy X-ray absorptiometry (DXA) with Hologic DXA equipment (Hologic Discovery W 81507) using the software for small animals. Results were obtained as grams of mineral content per square centimeter of bone area (g/cm2). The scanner was calibrated daily by in-house certified technician.

### 2.6. Blood Sample Preparation and Pretreatment

Samples were vortexed for 30 seconds; aqueous layer was transferred to 0.5 mL 3 KDa ultrafiltration filter (Millipore, USA). Filtrate was collected by centrifuging the sample at 13000 rpm for 45 mins. 350 *μ*L aqueous layer was transferred to a clean 2 mL centrifuge tube. 100 ul D2O and 50 *μ*L DSS standard solution (Anachro Technologies Inc, Canada) was added. Samples were mixed well before transfer to 5 mm NMR tube (Norwell, USA). Sample spectra were collected using a 600 MHz Bruker NMR spectrometer. MetNOESY sequence was used for its superior solvent suppression result. 100 ms mixing time along with a 990 ms presaturation was employed to match the acquisition parameters used in Chenomx Library. Spectra were collected at 25°C, with a total of 64 scans to obtain the required signal-to-noise ratio.

### 2.7. Urine Sample Preparation and Pretreatment

Samples were centrifuged at 13000 rpm for 2 mins; 540 *μ*L aqueous layer was transferred to a centrifuge tube. 60 *μ*L DSS standard solution (Anachro, Canada) was added. Samples were mixed for 10 seconds before being transferred to 5 mm NMR tube (Norwell, USA). Spectra were collected using the same protocol as described in Section 2.6. Because of lower concentration for most of the components, a total of 128 scans over a period of 15 min were used to acquire data for each sample.

### 2.8. Spectrogram Processing and Multivariate Pattern Recognition Analysis

The Free Induction Decay (FID) data was first processed using processor module in Chenomx NMR Suite 8.1. (Chenomx Inc., Edmonton, Canada). Briefly, data was automatically zero filled and underwent Fourier transform. The frequency domain data was then carefully phased and baseline corrected inside the same processor module. All spectra were referenced to DSS. Metabolites qualification and quantification were made by experienced analysts using Chenomx Compound Library. With the amount of 70 spectra, a total of 56 metabolites were identified and quantified. All metabolites' concentrations were used and normalized by Pareto scaling before multivariable analysis. R packages “pls” [19] and “ggplot2” [20] were used to perform PLS-DA analysis and plots, respectively.

### 2.9. Statistical Analysis

Data were expressed as means ± SD values. OGTT, ELISA, and fully automatic chemistry analyzer data analysis were performed by using GraphPad Prism 5 Software. One- or two-way ANOVA was performed to identify features with differential abundances across groups. Post hoc tests for the results were evaluated by Bonferroni test. *P* values less than 0.05 were considered statistically significant.

## 3. Results

### 3.1. ZJP Partially Inhibits the Increase of Blood Glucose Concentration in Diabetic Rats

**Figure 1:**
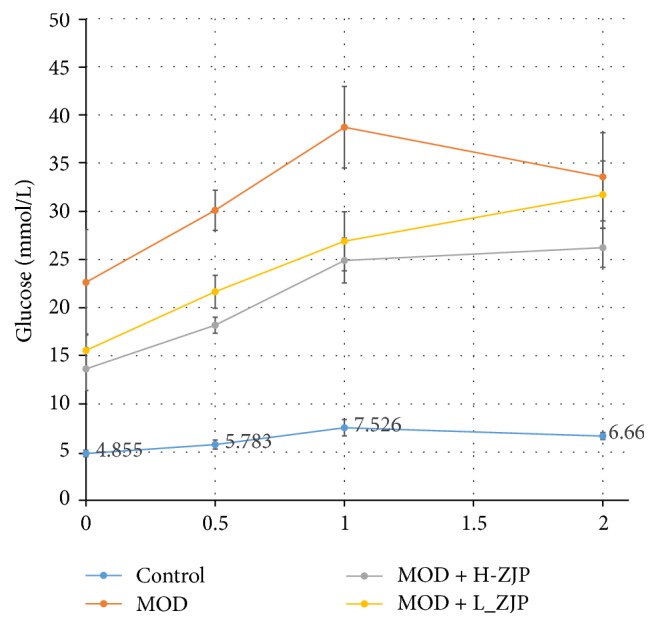
*Effect of ZJP on blood glucose concentration in diabetic rats. *The blood glucose concentrations were significantly increased after receiving glucose orally in MOD, MOD + H-ZJP, and MOD + L-ZJP compared with the control group; ZJP partially inhibits the increases of blood glucose concentration at the concentration of both 3.0 g/kg/d and 1.5 g/kg/d after 0.5 and 1.0 hours of glucose administration. ZJP partially inhibits the increases of blood glucose concentration at the concentration of both 3.0 g/kg/d after 2.0 hours of glucose administration.

As shown in Figure 1, the blood glucose concentrations were significantly increased after receiving glucose orally in MOD, MOD + H-ZJP, and MOD + L-ZJP compared with the control group (*P* < 0.001), while the increases in MOD group were partially inhibited by ZJP treatment at the dosage of both 3.0 g/kg/d and 1.5 g/kg/d after 0.5 hours and 1.0 hour of glucose administration (*P* < 0.001). The blood glucose concentration reached similar levels in MOD and MOD + L-ZJP groups after 2 hours of glucose administration (*P* < 0.001), while the blood glucose concentration remains at lower levels in MOD + L-ZJP groups compared with the control group (*P* < 0.001).

### 3.2. ZJP Improves Abnormal Glucose Metabolism in Diabetic Rats

**Figure 2:**
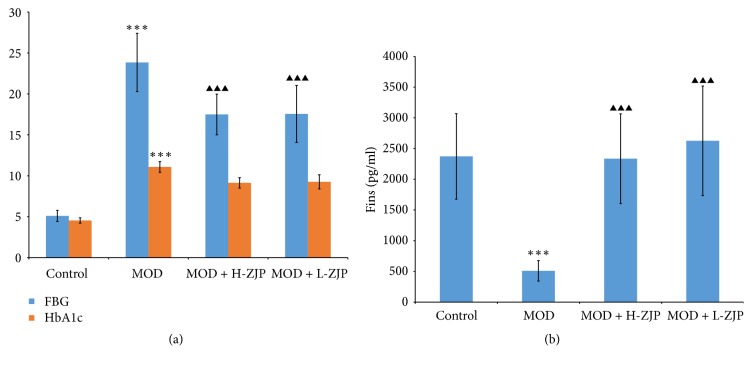
*Effect of ZJP on glucose metabolism in diabetic rats.* (a) The concentration of FBG and the percent of HbAlc were significantly increased in MOD group; ZJP partially inhibited the increases of FBG concentration but did not change the increased percent of HbAlc. (b) STZ significantly reduced the concentration of Fins; ZJP greatly inhibited the reduction of Fins concentration in diabetic rats. Asterisks indicate statistical significance (^*∗∗*^*P* < 0.01, ^*∗∗∗*^*P* < 0.001, control versus MOD). Triangle indicates statistical significance (^▲▲▲^*P* < 0.001, MOD versus MOD + H-ZJP or MOD + L-ZJP).

As shown in Figure 2(a), the concentration of FBG and the percent of HbAlc were significantly increased in MOD group, while the increase of FBG concentration was partially inhibited by ZJP at the dosage of both 3.0 g/kg/d and 1.5 g/kg/d (*P* < 0.001) and the percent of HbAlc was not changed with ZJP treatment (*P* > 0.05). As shown in Figure 2(b), STZ significantly reduced the concentration of Fins (*P* < 0.001), while ZJP completely inhibited the reduction induced by STZ (*P* < 0.001).

### 3.3. ZJP Partially Inhibits the Levels of BCa and P in Diabetic Rats

**Figure 3:**
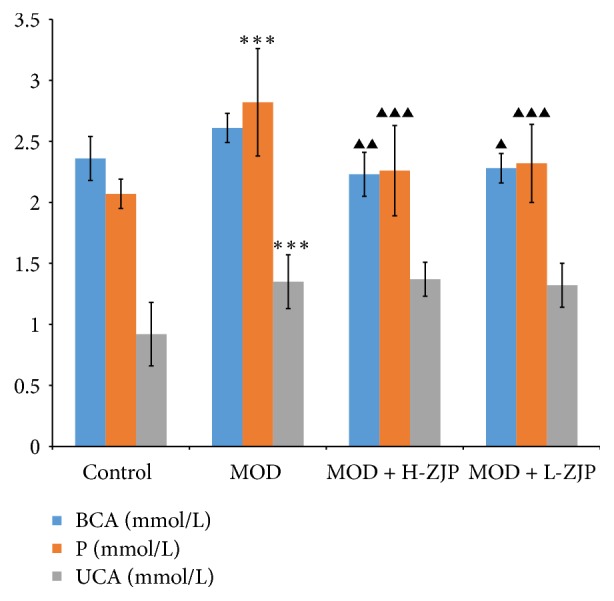
*Effect of ZJP on the levels of BCa, P, and UCa in diabetic rats.* STZ significantly increased the levels of BCa, P, and UCa in MOD group; ZJP partially inhibited the increases of BCa and P levels in diabetic rats but did not change the increase of UCa level. Asterisks indicate statistical significance (^*∗∗∗*^*P* < 0.001, control versus MOD). Triangle indicates statistical significance (^▲^*P* < 0.05, ^▲▲^*P* < 0.01, and ^▲▲▲^*P* < 0.001, MOD versus MOD + H-ZJP or MOD + L-ZJP).

As shown in Figure 3, STZ significantly increased the levels of BCa, P, and UCa in MOD group (*P* < 0.001), while the increase of BCa level was partially inhibited by ZJP at the dosage of both 3.0 g/kg/d (*P* < 0.01) and 1.5 g/kg/d (*P* < 0.05) and the increase of P level was also partially inhibited by ZJP at the dosage of both 3.0 g/kg/d (*P* < 0.001) and 1.5 g/kg/d (*P* < 0.001). However, the increase of UCa in diabetic rats was not changed by ZJP administration (*P* > 0.05).

### 3.4. ZJP Improves Abnormal Bone Metabolism in Diabetic Rats

**Figure 4:**
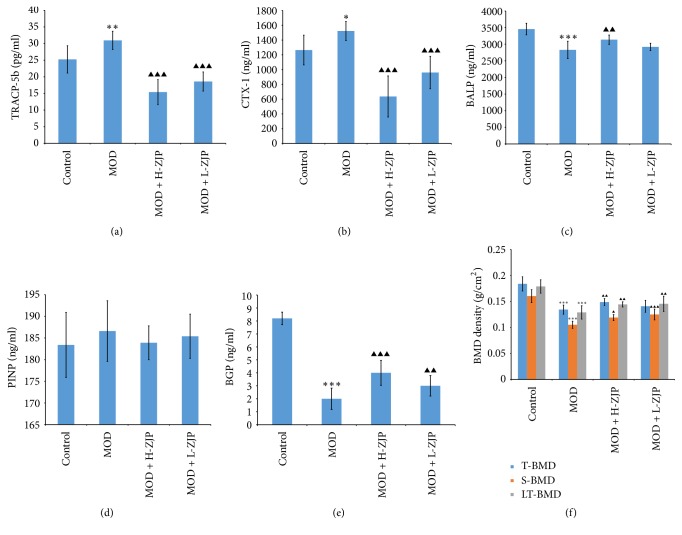
*Effect of ZJP on bone metabolism in diabetic rats.* (a) STZ significantly increased the level of TRACP-5b in MOD group; ZJP completely inhibited the increase of TRACP-5b level in diabetic rats. (b) STZ significantly increased the level of CTX in MOD group; ZJP completely inhibited the increase of CTX levels in diabetic rats. (c) STZ significantly decreased the level of BALP in MOD group; ZJP attenuates the reduction of BALP level at the concentration of 3.0 g/kg/d in diabetic rats. (d) The level of PINP was not changed by STZ and ZJP administration. (e) STZ significantly decreased the level of BGP in MOD group; ZJP attenuates the reduction of BGP level in diabetic rats. (f) STZ significantly decreased the T-BMD, S-BMD, and LT-BMD; ZJP partially inhibited the reduction of T-BMD, S-BMD, and LT-BMD in diabetic rats (^*∗*^*P* < 0.05, ^*∗∗*^*P* < 0.01, and ^*∗∗∗*^*P* < 0.01, control versus MOD). Triangle indicates statistical significance (^▲^*P* < 0.05, ^▲▲^*P* < 0.01, and ^▲▲▲^*P* < 0.001, MOD versus MOD + H-ZJP or MOD + L-ZJP).

TRACP-5b and CTX are normally used to diagnose osteoporosis, malignant bone tumours, or other pathology and to monitor antiresorptive therapy, which indicate the changes of bone resorption most satisfactorily [21, 22]. As shown in Figures 4(a) and 4(b), STZ significantly increased the levels of TRACP-5b (*P* < 0.01) and CTX (*P* < 0.05) in MOD group, while the increase of TRACP-5b level was completely inhibited by ZJP at the concentration of both 3.0 g/kg/d (*P* < 0.001) and 1.5 g/kg/d (*P* < 0.001), and the increase of CTX-1 level was also completely inhibited by ZJP at the dosage of both 3.0 g/kg/d (*P* < 0.001) and 1.5 g/kg/d (*P* < 0.001). Serum BALP, PINP, and BGP have emerged as reliable markers of bone turnover in humans and is routinely used to monitor bone formation [23, 24]. As shown in Figure 4(c), the level of BALP was significantly decreased by STZ (*P* < 0.001), while ZJP attenuates the reduction at the concentration of 3.0 g/kg/d (*P* < 0.01). As shown in Figure 4(d), the level of PINP was not changed by STZ and ZJP administration (*P* > 0.05). As shown in Figure 4(e), the level of BGP was significantly decreased in diabetic rats (*P* < 0.001), while ZJP attenuates the reduction of BGP at the concentration of 3.0 g/kg/d (*P* < 0.01) and 1.5 g/kg/d (*P* < 0.01). As shown in Figure 4(f), the T-BMD, S-BMD, and LT-BMD were significantly decreased by STZ (*P* < 0.001), while the reduction of T-BMD was partially inhibited by STZ at the concentration of 3.0 g/kg/d (*P* < 0.001). Meanwhile, the reduction of S-BMD was partially inhibited by STZ at the concentration of both 3.0 g/kg/d (*P* < 0.05) and 1.5 g/kg/d (*P* < 0.001), and reduction of LT-BMD was also partially inhibited by STZ at the concentration of both 3.0 g/kg/d (*P* < 0.01) and 1.5 g/kg/d (*P* < 0.01).

### 3.5. ^1^H-NMR Based Metabolomics Study on Blood of Diabetic Rats

**Figure 5:**
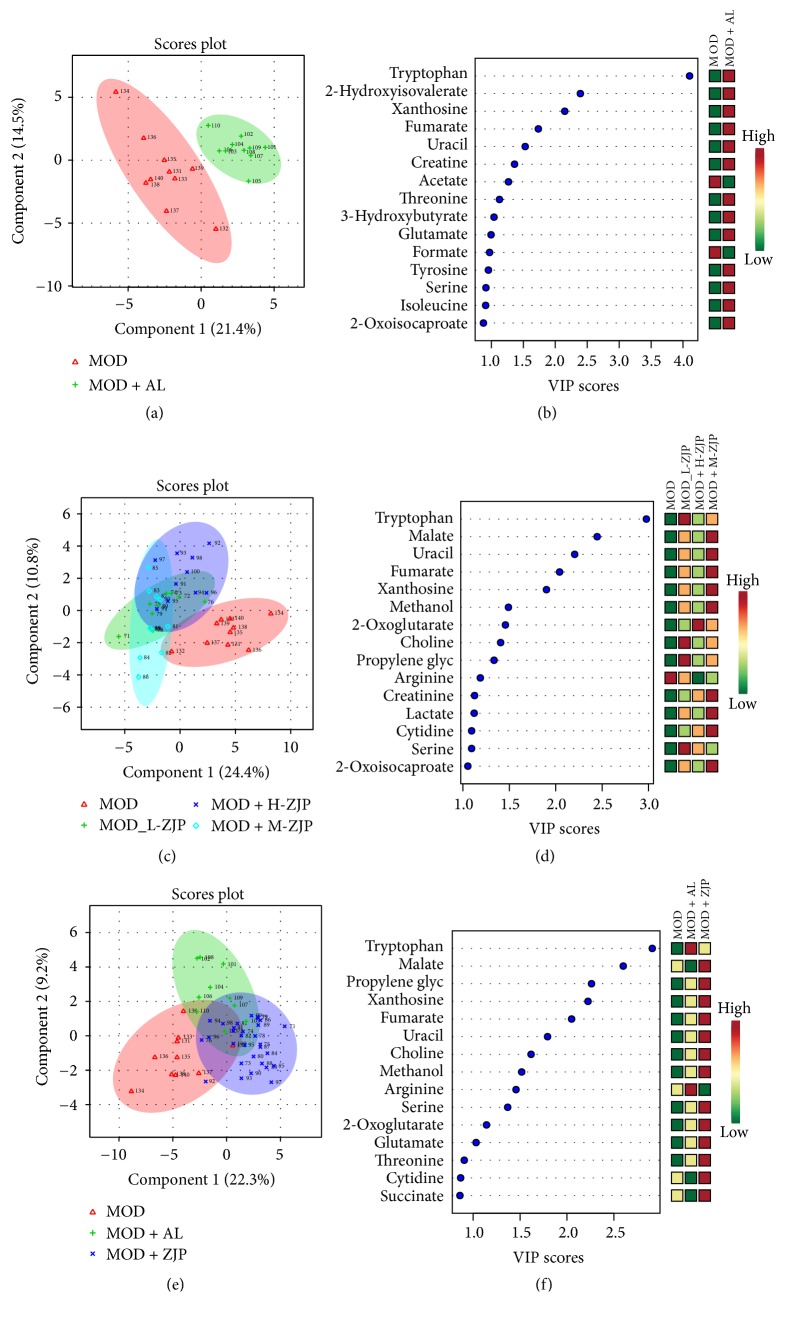
*Multivariate data analysis of blood metabolomics.* (a) The metabolic state of MOD and CON groups. (b) VIP scores of MOD and CON groups. (c) The metabolic state of MOD, MOD + H-ZJP, MOD + L-ZJP, and CON groups. (d) VIP scores of MOD, MOD + H-ZJP, MOD + L-ZJP, and CON groups. (e) The metabolic state of MOD and MOD + H-ZJP groups. (f) VIP scores of MOD and MOD + H-ZJP groups.

By using the targeted profiling method, the metabolites were quantified and qualified using Chenomx NMR Suite 8.0 by an experienced lab technician. All identified metabolites were used for multivariable analysis. As shown in Figure 5(a), PLS-DA was used to bring out the specific variation in the blood samples of MOD and CON groups. In the PLS-DA score plot, the metabolic state of MOD group was significantly different from the CON, indicating that diabetes changed the endogenous substances metabolism and significantly altered the metabolic fingerprints of rat blood. A Variable Importance in Projection (VIP) plot in which the metabolites were ranked by their contribution to distinguishing the cases of diabetes from CON group is shown in Figure 5(b). As shown in Figure 5(c), in the PLS-DA score plot, the metabolic state of MOD group was significantly different from the MOD + H-ZJP and MOD + L -ZJP groups, indicating that different concentrations of ZJP can change metabolic fingerprints of rat blood, suggesting that ZJP played a therapeutic role in diabetic rats. A VIP plot in which the metabolites were ranked by their contribution to distinguishing the cases of diabetes from ZJP treated groups is shown in Figure 5(d). As shown in Figure 5(e), in the PLS-DA score plot, the metabolic state of MOD group was significantly different from the MOD + H-ZJP groups, and the metabolic state of MOD + H-ZJP group was different from the MOD group, indicating that ZJP played a therapeutic role in diabetic rats. A VIP plot in which the metabolites were ranked by their contribution to distinguishing the cases of diabetes from ZJP is shown in Figure 5(f).

### 3.6. ^1^H-NMR Based Metabolomics Study on Urine of Diabetic Rats

**Figure 6:**
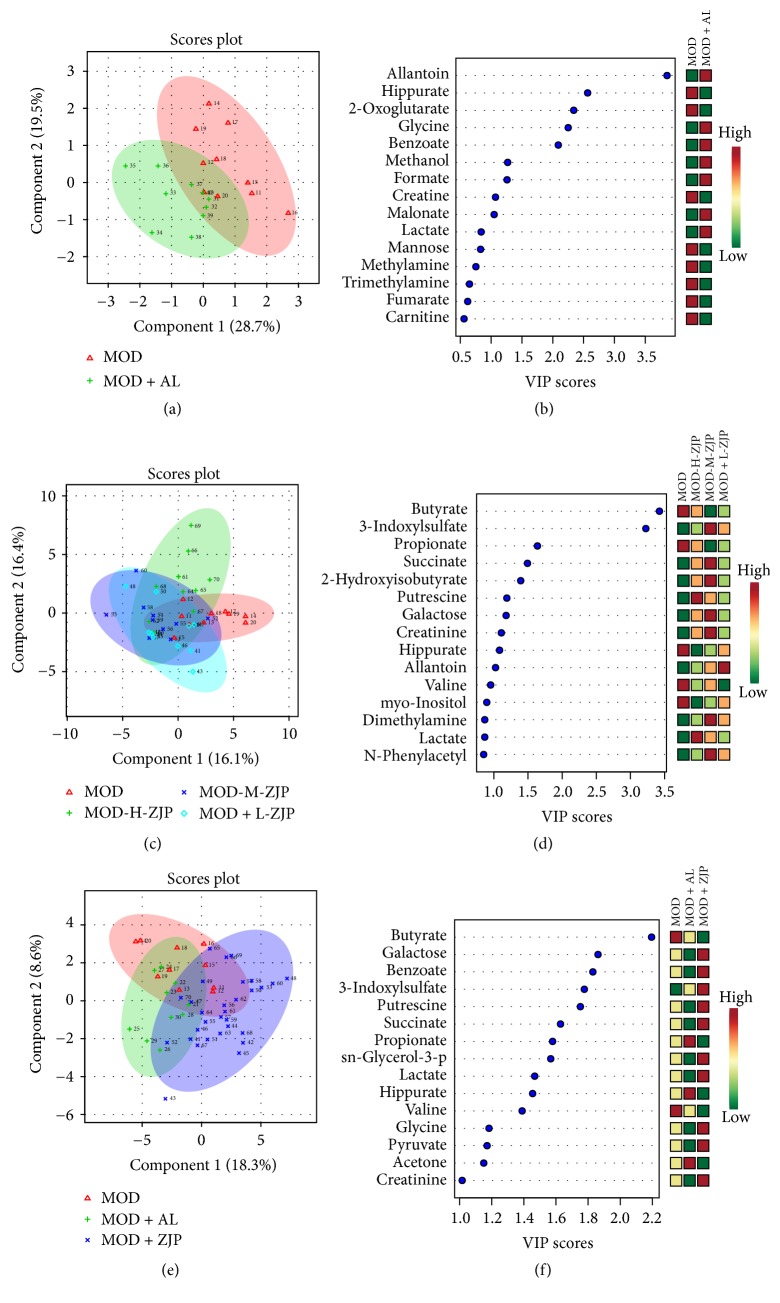
*Multivariate data analysis of urine metabolomics.* (a) The metabolic state of MOD and CON groups. (b) VIP scores of MOD and CON groups. (c) The metabolic state of MOD, MOD + H-ZJP, MOD + L-ZJP, and CON groups. (d) VIP scores of MOD, MOD + H-ZJP, MOD + L-ZJP, and CON groups. (e) The metabolic state of MOD and MOD + H-ZJP groups. (f) VIP scores of MOD and MOD + H-ZJP groups.

PLS-DA was used to bring out the specific variation in the urine samples of MOD and CON groups. As shown in Figure 6(a), in the PLS-DA score plot, the metabolic state of MOD group was significantly different from the CON, indicating that diabetes changed the endogenous substances metabolism and significantly altered the metabolic fingerprints of rat urine. A VIP plot in which the metabolites were ranked by their contribution to distinguishing the cases of diabetes from CON group is shown in Figure 6(b). As shown in Figure 6(c), in the PLS-DA score plot, the metabolic state of MOD group was significantly different from the MOD + H-ZJP and MOD + L-ZJP groups, indicating that different concentrations of ZJP can change metabolic fingerprints of rat urine. A VIP plot in which the metabolites were ranked by their contribution to distinguishing the cases of diabetes from ZJP treated groups is shown in Figure 6(d). As shown in Figure 5(e), in the PLS-DA score plot, the metabolic state of MOD group was significantly different from the MOD + H-ZJP and CON groups, and the metabolic state of MOD + H-ZJP group was different from the MOD group. A VIP plot in which the metabolites were ranked by their contribution to distinguishing the cases of diabetes from ZJP groups is shown in Figure 6(f).

## 4. Discussion

Diabetic osteoporosis (DO), characterized by low bone mass, is a common complication of diabetes but asymptomatic in diabetic patients until the fracture [10]. It has been reported that fracture risk in T2DM patients with poor glycemic control increased by 47%–62% compared with nondiabetic patients and those with good glycemic control [25]. The basic mechanism underlying the development of osteoporosis is the bone-remodeling imbalance [26]. The characteristics of bone reconstruction associated with DO are thought to include a continuous increase in the number and activation level of osteoclasts with a stable proliferation and activity of osteoblasts [27, 28]. In previous epidemiological studies, diabetic patients show moderately increased risk for osteoporotic bone fractures compared to general population [29]. However, the underlying molecular mechanism causing osteoporosis in diabetes remains unclear. Numerous research studies are currently focusing on the effects of traditional Chinese medicine formulations on osteoporosis [30, 31].

In the present study, we investigate the effect of the traditional Chinese medicine ZJP on STZ-induced DO rat. We first examined the glucose tolerance in diabetic rats by using the OGTT; the result showed that ZJP significantly decreased the blood glucose level in diabetic rats compared with the MOD group (Figure 1), indicating that ZJP may exert a role in diabetes induced by STZ. In support of this, previous study has demonstrated that vildagliptin also elicited decrease in glucose during OGTT in patients with T2DM [32]. We further investigated the glucose metabolism in diabetic rats. We examined the levels of glucose metabolism markers including FBG, HbAlc, and Fins. Results showed that ZJP decreased the level of FBG and increased Fins level in diabetic rats but has no effect on HbAlc level (Figure 2). Generally, as the important glucose metabolism indexes, the levels of FBG and HbAlc were significantly higher, and the level of Fins was significantly lower in diabetic patients than that of nondiabetic patients. Studies showed that FBG and HbAlc levels were downregulated, and the level of Fins was upregulated in diabetic patients after drug treatment [33–35]. However, ZJP has no effect on HbAlc level in our study. This phenomenon is probably due to the fact that HbAlc is an indicator of long-term blood glucose control, whereas our experiment period is only 8 weeks. Moreover, once-daily treatment of ZJP cannot last 24 hours, which led blood glucose level to be fluctuated. Besides, we also examined the calcium-phosphorus metabolism in diabetic rats, and the results demonstrated that the levels of BCa, P, and UCa were also increased in diabetic rats, while the increase of BCa and P levels was partially inhibited by ZJP. However, the level of UCa was not changed in diabetic rats treated with ZJP (Figure 3). We next examined the bone metabolism to detect osteoporosis in diabetic rats. The bone resorption indexes TRACP-5b and CTX, as well as the bone formation indexes BALP, PINP, BGP, and BMD, were analyzed. Data showed that ZJP inhibited the increases of TRACP-5b and CTX levels (Figures 4(a) and 4(b)) and attenuated the reduction BALP and BGP levels (Figures 4(c) and 4(e)) in diabetic rats compared with the MOD group but did not change the level of PINP. Moreover, the T-BMD, S-BMD, and LT-BMD were also partially recovered by the treatment of ZJP (Figure 4(f)). These data supported that diabetes induced the formation of osteoporosis in rats, while treatment of ZJP markedly attenuated the development of osteoporosis.

Moreover, we further investigated the metabonomics of blood and urine in diabetic rats to determine the therapeutic role of ZJP on diabetic rats which has been identified with osteoporosis. Result showed that diabetes changed the endogenous substances metabolism and significantly altered the blood metabolic fingerprints of rats (Figure 5(a)). The levels of acetate, urea, acetone, and citrulline were significantly increased in the blood of DO rats (Figure 5(b)). Treatment with ZJP (H-ZJP, L-ZJP) significantly altered the endogenous substances metabolism and the metabolic fingerprints of rat blood (Figure 5(c)). The levels of acetate, urea, acetone, and citrulline are significantly decreased in the blood of DO rats after ZJP treatment (Figure 5(d)). These data suggested that ZJP might exert a therapeutic effect on DO rats.

Taken together, our results demonstrate that ZJP can effectively improve glucose metabolism through regulation of FBG and Fins, improving abnormal bone metabolism, such as inhibiting excessive bone absorption and promoting the reduced bone resorption through regulation of TRACP-5b, CTX, BALP, and BGP, enhancing BMD, and improving blood and urinary metabolism in DO rats. Our findings suggest that ZJP may be a potentially effective medicine for the treatment of DO.
